# Gender Division of Labor, Burnout, and Intention to Leave Work Among Young Female Nurses in Japan: A Cross-Sectional Study

**DOI:** 10.3390/ijerph16122201

**Published:** 2019-06-21

**Authors:** Sachiko Minamizono, Kyoko Nomura, Yuki Inoue, Haruko Hiraike, Akiko Tsuchiya, Hiroko Okinaga, Jan Illing

**Affiliations:** 1 Department of Public Health, Akita University Graduate School of Medicine, 1-1-1Hondo, Akita City 010-8543, Japan; sachikot@med.akita-u.ac.jp; 2 Support Center for Women Physicians and Researchers, Teikyo University, 2-11-1Kaga, Itabashi-ku, Tokyo 173-8605, Japan; hiokinaga@gmail.com; 3 Funabashi Municipal Medical Center, 1-21-1 Kanasugi, Funabashi City, Chiba 273-8588, Japan; inoueyuki112@gmail.com; 4 Department of Obstetrics and Gynaecology, Teikyo University School of Medicine, 2-11-1Kaga, Itabashi-ku, Tokyo 173-8605, Japan; haruko.hiraike@gmail.com; 5 Nursing Department, Teikyo University Hospital, 2-11-1Kaga, Itabashi-ku, Tokyo 173-8605, Japan; a-tutiya@med.teikyo-u.ac.jp; 6 School of Medical Education, Newcastle University, Newcastle upon Tyne, NE2 4HH, UK; Jan.Illing@newcastle.ac.uk

**Keywords:** burnout, gender division of labor, intention to leave, job stress, nurse, support, work-family conflict

## Abstract

Women in Japan face difficulties balancing work and personal life due to the gender division of labor, and medical professions are no exception. The purpose of this study was to investigate if the gender division of labor affects the intention to leave the workplace among the nursing profession. Among 328 female nurses working for three university-affiliated hospitals in Tokyo, Japan, above 70% were in their 20s and 30s and single, and agreed with the gender division of labor that men should be the breadwinner and women should assume family responsibilities. Adjusting for three types of Copenhagen burnout inventory, stepwise multivariable logistic regression models identified that being younger (all *p*-values < 0.05), each domain of burnout score (each *p* < 0.001 for work-, personal-, and client-related burnout) increased a risk of intention to leave, and high support decreased the risk (all *p* < 0.001). Women who agreed with the gender division of labor were more likely to have intentions to leave (*p* = 0.003 but this association disappeared when adjusted. The findings of study demonstrate that perceptions toward gender division of labor are not a determinant of intention to leave the workplace but the young nurses and those who scored high on burnout were the most vulnerable population.

## 1. Introduction

The retention of newly certified nurses is important for organizations in terms of cost and time commitment for human resources management [1]. Health care facilities invest thousands of dollars in recruiting and training nurses [2,3]. However, there is an international crisis, as newly graduated nurses are leaving their first place of employment within their first year, having enormous financial implications [3,4,5,6]. Therefore, it is necessary to improve the working environment for long-term nurse retention resulting in good quality of patient care [7,8]. 

Nursing is a profession that requires face-to-face contact with patients and their families, and nurses encounter patient suffering and deaths on regular basis. As a result of these negative experiences, newly graduated nurses may experience a reality shock, especially within their first year of employment [9,10]. Discrepancies between what they were taught at university and the reality of nursing causes stress and shock for new nurses [11]. Burnout and job dissatisfaction have been found to increase turnover and reduce retention [12]. A preceptorship, known as one-to-one pairing, involves an experienced nurse (nurse preceptor) supervising a less experienced nurse to provide individualized support and teaching [13]. In the setting of teaching hospitals, where the majority of nurses are younger, this places a heavy burden on the preceptor, and particularly on an inexperienced preceptor who is also young [14,15]. In this case, young and inexperienced nurses became a high-risk group for psychological burnout as the demands of the job have increased [6,12]. In Japan, younger nurses tend to experience high levels of burnout [16]. Such high levels of burnout in younger workers could be due to this group having less professional experience and thus not having sufficient time to formulate effective strategies for dealing with occupational stress [6,17].

According to a governmental survey [18], the most frequent reason given by nurses for leaving their job is “life events” such as pregnancy and childrearing. In Japan, stereotypical gender roles—that men should be the breadwinners and women should assume family responsibility—is prevalent (nearly 40%) even among the younger generation in their 20s [19], and is deeply rooted in the mindset of the Japanese population. Previously, we revealed that the idea is also prevalent even among female medical doctors [20]. Consequently, Japanese female medical doctors tend to stop working at the time of childbirth/rearing, which threatens the physician workforce supply in Japan. Short periods of staff retention in the workplace may deprive women of the opportunity for promotion to a leadership position in their profession and may account for the high gender gap index of Japan, which ranks 114 among 144 countries [21].

Previous literature on nurse retention has identified that working conditions, individual characteristics including marital status, and having children influences staff retention [7,22,23,24,25]. However, few studies have explored perceptions towards the gender division of labor deeply embedded in mindset of society. Hence, the purpose of this study was to investigate if gender division of labor affects intention to leave the workplace and to identify factors including burnout associated with the intension among the nursing profession in Japan. 

## 2. Materials and Methods 

### 2.1. Participants

**Figure 1:**
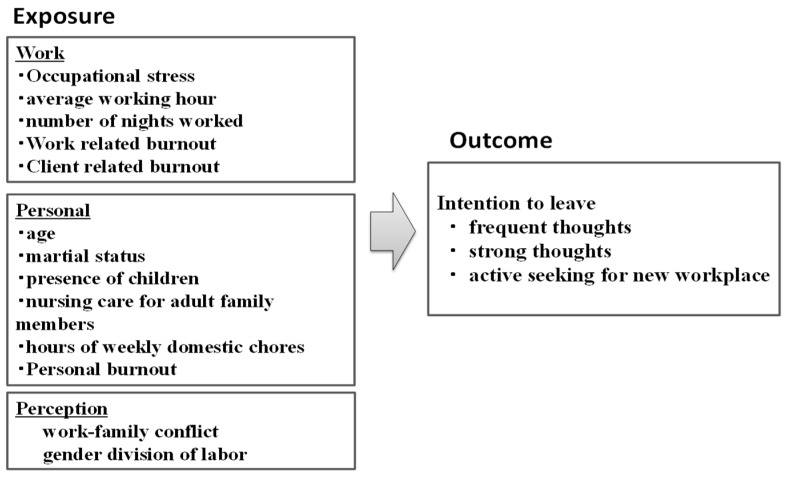
The theoretical framework and hypothesis of this study. Conceptual framework for nurse’s intention to leave the workplace.

This cross-sectional study used secondary data from a survey of work-family conflict among workers at a large private medical university that has five campuses and three affiliated hospitals. In February 2016, a total of 3493 employees, including 1698 nurses, were invited to participate in the study by mail and 1186, including 441 nurses, provided informed consent and returned the self-administered questionnaires on an anonymous and voluntary basis (overall response rate 34%, response rate for nurses 26%). Inclusion criterion was female nurses. Exclusion criteria included faculty members (*n* = 521), other medical personnel (*n* = 196), male staff (*n* = 34), and missing values on every single variable investigated (*n* = 113). This left 328 nurses for analysis. Referring to previous studies that investigated nurse retention, we conceptualized the theoretical framework in Figure 1 [23,24,25]. For factors for intention to leave at workplace, we collected information about work and personal characteristics, and the perceptions of work-family conflict and gender division of labor. This study was approved by the Teikyo University ethics committee (No.13-1310).

### 2.2. Measures 

The questionnaire items addressed personal factors (age, marital status, caring responsibility of children or adult family members, hours spent doing weekly and weekend domestic chores, personal burnout), work environment characteristics (occupational stress, years in employment, daily working hours, number of nights worked per month, job strain, job support, work related burnout, client related burnout), individual perceptions (perceptions toward gender division of labor and work-family conflict), and intention to leave (frequent thoughts, strong thoughts, and actively seeking new work place). Regarding job strain and job support, we used the Job Contents Questionnaire (JCQ). Burnout was measured by the Copenhagen burnout inventory.

#### 2.2.1. Intention to Leave

“Intention to leave” is known as an independent predictor of actual employee retention [26,27] and thus we used this intention as the outcome of interest. Referring to a previous study [28],intention to leave was defined if any of the following three questions received a positive response: (1) frequent thoughts of intention to leave (i.e., I often think of leaving work place); (2) strong thoughts of intention to leave (i.e., I’ll leave my work place if I can); and (3) actively seeking a new workplace (i.e., I am actively seeking a new workplace). Responses were based on a Likert scale from strongly disagree (1) to strongly agree (4) and subsequently divided into binary, disagree, or agree.

#### 2.2.2. Gender Division of Labor

Gender division of labor was measured based on the following three items that were used for a previous Cabinet Survey [19]: (1) men are expected to fulfill traditional male roles (e.g., breadwinner) and women assume family responsibilities (e.g., household chores); (2) as a woman, being a wife or a mother is more important than work; and (3) for a mother, engagement in childrearing is the first priority. Responses can range from 1 for strongly disagree to 5 for strongly agree. Affirmative perceptions toward gender division of labor are defined when a larger than median summation of these three responses (binary variable) is provided.

#### 2.2.3. Work-Family Conflict, Burnout, and Occupational Stress

To measure work-family conflict, we selected the following four questions referring to a previous study [29]: (1) My job occupies lots of my time, which makes it difficult to fulfil family duties; (2) the amount of time required for family responsibilities interferes with my work productivity; (3) I am often too exhausted to do anything for my family when I come back from work; and (4) due to psychological burden resulting from my family duties, I often find it difficult to focus on work. Responses were based on a Likert scale from strongly disagree (1) to strongly agree (5). Work-family conflict is defined if summation of (1) and (3), and that of (2) and (4) is higher than the median (binary variable). 

We used the Japanese version of the Copenhagen burnout inventory in this study. The Copenhagen burnout inventory examines exhaustion and its attribution to three distinctive aspects: personal, work-related, and client-oriented burnout, and the three scales predict future sickness absence and intention to quit [30]. Personal burnout (PBO) pertains to general symptoms of physical or mental exhaustion, which are not always related to a given particular situation in the work environment and applies to everyone. Work-related burnout (WBO) pertains to symptoms of exhaustion that are related to the work of the subject and applies to everyone in the workforce. Client-related burnout (CBO) pertains to symptoms of exhaustion related to the subject’s work with clients and applies to employees in human service work such as nurses and teachers. Thus, “client” can be replaced with “patients” in this study. A total of 19 items were scored on a five-point Linkert scale. The three burnout scores (continuous variable) were calculated by averaging all relevant items, so that higher scores indicate a higher degree of burnout. 

Occupational stress has been evaluated by the Job Content Questionnaire (JCQ) based on Karasek’s Demand-Control Model [31,32]. The Japanese version of the JCQ is considered to be reliable and valid for assessing job stressors among Japanese employees [33]. This instrument includes scales for job demands, job control, and worksite social support using a Likert Scale format, varying from 1 (strongly disagree) to 4 (strongly agree). Job strain is computed by dividing scores for job demand divided by job control’s scale of JCQ and divided into binary variables above and below 75% (i.e., 0.54) of its distribution. Job support was also divided into binary variable with a median (i.e., 24).

### 2.3. Data Analysis

According to age group category, intention to leave was analyzed with all relevant variables using chi-square tests for categorical variables and one-way analysis of variance for contentious variables. A logistic regression model was used to investigate influence of each factor on intention to leave. Odds ratio (OR) was computed along with 95% confidence intervals (95% CIs). Multivariable stepwise regression models were used to identify factors associated with intention to leave for each Copenhagen burnout inventory domain. Significance was determined as 5%. All analyses were performed using SAS version 9.4 (SAS Institute Inc., Cary, NC, USA).

## 3. Results

Table 1 indicates age group differences in the items investigated. In total, 76% were in their 20s and 30s (mean, 32 years old) and 69% were single, about 70% admitted work-family conflict and 66.7% of those in their 20s agreed with the gender division of labor. Burnout scores of all three domains and proportions of JCQ support and intention to leave were highest in group of 20-year-olds, although more than half (56.8%) of women in their 20s received high support. 

Table 2 indicates that women who are in their 20s (*p* = 0.006), who agreed with the concept of gender division of labor (*p* = 0.003), who had longer working hours (*p* = 0.038), who had high levels of strain (*p* = 0.010), and who had higher scores of three domains (all *p* > 0.001) were more likely to report intentions to leave work. Those with a child (*p* = 0.005) and high levels of support in the workplace (*p* < 0.001) were less likely to report intentions to leave the workplace. Although not reaching significance, nurses who had four or more working nights per month compared with nurses who had three or less working nights tended to have intention to leave (*p* = 0.097).

Table 3 indicates the results of the univariate logistic regression model for the factors associated with intention to leave. Significant factors that increased a risk of intention to leave included being from the younger generation (20s) (OR 2.55, 95% CI: 1.43–4.54), agreement with the concept of gender division of labor (OR 2.14, 95% CI: 1.30–3.53), high job strain (OR 1.91, 95% CI: 1.16–3.15), and a 10-unit increase in three domains of the Copenhagen burnout inventory (OR 1.67, 95% CI: 1.42–1.97 for WBO, OR 1.42, 95% CI: 1.25–1.62 for PBO, OR 1.51, 95% CI: 1.30–1.76 for CBO). Presence of a child (OR 0.48, 95% CI: 0.28–0.80) and the presence of high levels of support (OR 0.30, 95% CI: 0.18–0.52) significantly decreased the risk of intention to leave the workplace.

Table 4 shows the result of the stepwise multivariable logistic regression model to identify factors associated with intention to leave according to the three domains of the Copenhagen Burnout Inventory. Significant factors identified included age, job support, and the three domains of the Copenhagen Burnout Inventory: younger generation (OR 2.37, 95% CI: 1.22–4.61 for WBO, OR 2.85, 95% CI: 1.49–5.46 for PBO, OR 3.31, 95% CI: 1.72–6.38 for CBO), high support (OR 0.33, 95% CI: 0.18-0.60 for WBO, OR 0.28, 95% CI: 0.16–0.52 for PBO, and OR 0.30, 95% CI: 0.16–0.55 for CBO), and higher burnout scores (OR 1.58, 95% CI: 1.32–1.89 for WBO, OR 1.39, 95% CI: 1.20–1.60 for PBO, and OR 1.47, 95% CI: 1.24–1.73 for CBO). The interaction among age, JCQ support, and the three domains did not reach statistical significance.

## 4. Discussion

In this study of Japanese female nurses working for three university affiliated hospitals, we investigated factors associated with intention to leave the workplace. Of particular relevance was perceptions toward the gender division of labor. After adjusting for the working and personal life conditions of individuals, we identified that women in their 20s and those who had higher burnout scores had an increased risk of intention to leave, supporting the international literature on the high turnover of newly qualified nurses [6,7,34], whereas job support provided protection and decreased this risk. We found that perceptions on the gender division of labor and work-family conflict were not associated with intention to leave a workplace among female nurses in this university setting. 

In this study, agreeing with the gender division of labor did not influence intention to leave. According to the Cabinet Survey of Japan [19], more than half of women agreed that the mother should stay home when a child is young. This idea is widely accepted by older Japanese people, believing that children need their mothers to be disciplined well. Women are more likely to apply for parental leave, but very few men do [35]. Women who agree with gender division of labor have long been thought to tend to quit working following life events such as marriage, pregnancy, or child rearing. However, this present study demonstrated that upholding views about the importance of gender division of labor are not in conflict with continuous employment and career development and do not increase the risk of intention to leave the workplace among nurses. Alternatively, as the majority of the respondents in our study sample were not married, our subjects may not yet have family responsibilities. If the majority of our subjects had children and family responsibilities, their agreement with gender division in labor might have had a different effect on intention to leave a workplace from that observed in the present study, but the direction of this potential bias may not be clear and may or may not result in random error.

The literature suggests that work-family conflict may be a real obstacle for women to continuously work [24,36,37].

The discrepancy between the non-significant result for work-family conflict in our study and previous studies could be explained by Yamaguchi et al. recruiting older nurses. This suggests that being older may increase the perception of increased work-family conflict. The majority of our study population was not married, indicating that they were less likely to experience work-family conflict, although 78% of our subjects perceived that they had work-family conflict. According to the labor force survey of the Japanese government, the age-stratified labor participation rate declines in the late 20s, and again in the early in their 40s, when women bear and raise children. This hiatus is called an M-curve, suggesting that women in these two age bands face work-family conflicts [38]. Our sample population may be younger than these age generations, suggesting high percentages of self-reported work-family conflict may reflect a psychological response to an as-yet unrealized fear of the future. Literature in this area of research reported that it is generally challenging for novice nurses to have an accurate perspective of their profession and to obtain job satisfaction [39] and thus it is important to support them during the process of acquiring specialized skills in nursing [40]. Although we cannot be sure if the reported high percentage of work-family conflict does reflect a psychological response to unconscious fear toward the future profession, we should explore this further together with the significance of being younger in age and the increased risk of intention to leave. 

This study demonstrated that young female nurses are more likely to leave the workplace, supporting the international literature highlighting a high turnover of newly qualified nurses [6,7,34]. We also identified that the average daily working hours were the longest and number of nights worked per month was highest for nurses in their 20s. These two working conditions were not selected in the final logistic model but univariate analyses identified that these two conditions significantly increased the risk of intention to leave the workplace. Our additional analyses demonstrated that working hours and the number of on-calls became nonsignificant when adjusting for age. Thus, working conditions on their own were not associated with intention to leave, but age was a strong determinant. This indicates that young nurses, like student nurses, are less resilient to stress than experienced nurses. Previously, a Japanese study on nurses reported that individual nurse burnout, dissatisfaction, and poorer quality of care were associated with workplaces that have a larger percentages of inexperienced nurses [41]. Studies on student nurses showed they are more affected than older nurses by workplace violence experiences, report more distress [42], and that distressed student nurses learn less than their colleagues [43]. The state of distress, in turn, exposes nurses to workplace violence [44,45]. Psychosocial factors and violence are important factors for withdrawal, and future studies should be undertaken in this direction. Thus, for inexperienced young nurses, their retention at work could be improved if they had more support from seniors, were trained further on practical skills and on how to adapt themselves to work efficiently, and if long working hours are avoided.

We demonstrated that social support from seniors or colleagues did have a protective role for those who reported an intention to leave the workplace. The hierarchy amongst nurses is supported and nurses are ranked by their level of education and licensure, as well as years of experience. Therefore, inexperienced young nurses may tend to be left alone at work. In addition to having preceptors present in the nurse education system, a mentor and an experienced and trusted external adviser could be used as social support for young female nurses to avoid their leaving the workplace so soon after qualification. Our previous study identified that a consultation service offered by an external body, such as the section for diversity, was effective in reducing burnout among medical faculties [46]. Two earlier studies about nurses in Japan reported that nurses who experience high levels of job control or work engagement (protective indicators) tend to be retained [24,25]. However, nurses in these two studies were much older than those in our sample, which may account for the insignificant findings of job control in our study. In addition, two previous nurse studies had different research questions from ours, focusing more on experienced nurses: one study focused on characteristics of workplace including hospital, home health care and nursing home, and the other study investigated long-term care nurses. 

A strength of this study was that we were able to investigate both individual factors of work and private life. Furthermore, this study enabled us to evaluate how the perceptions of gender division of labor affect decision-making about continuing employment among female nurses. Finally, despite the small sample size, the majority of our subjects were young, single, and new graduates from nursing schools, and therefore we were able to investigate factors associated with intention to leave particularly among the young female nurse profession. There are several limitations to address in addition to the small sample size. First, this study used a self-reported survey; therefore, it is possible that those who did not participate may or may not face a high degree of burnout. Second, our target population of nurses did not include male nurses. Recently, the number of male nurses is increasing and we do not know if they experience more psychological stress working in a woman-dominated job. Future studies on nurse retention should therefore include male nurses. Third, we did not collect information about life events that cause psychological stress. Burnout measured in this study may be influenced by such psychological events. Fourth, we did not measure organizational factors. Previously, we suggested that organizational climate with gender equity mitigates burnout among university academics [46]. Despite these limitations, we demonstrated that those who were young and perceived burnout were more likely to leave the workplace, but the presence of social support reduced the risk of the intention to leave the workplace. In addition, we confirmed that the perception of the gender division of labor was not associated with intention to leave the workplace, suggesting that agreement with the gender division of labor did not harm the decision to continue work.

## 5. Conclusions

This study found that perceptions toward the gender division of labor were not associated with intention to leave the workplace, or rather the young generation (in their 20s) are the most vulnerable population to quitting working. The idea of the gender division of labor is respected in the Japanese mindset as “Good Wives and Wise Mothers”. The result of this study suggests that the gender division of labor does not conflict with work continuation. From the perspective of Human Resource Management, the retention of newly certified nurses is key to running medical institutions. This study confirmed that nurses who experience burnout were more likely to intend to leave work, which is consistent with previous studies. Social support from senior and colleague including preceptorship, mentorship, and consultation services at the work place would be beneficial for young female nurses.

## Figures and Tables

**Table 1:** Characteristics according to age group (*N* = 328).

Variables	20–29 (*N* = 183)		30–39 (*N* = 66)		40–49 (*N* = 53)		50– (N = 26)		*p*
	*N*	%	*N*	%	*N*	%	*N*	%	
Marital status									<0.001
Married	24	13.1	33	50.0	32	60.4	13	50.0	
Single (Including Divorced or Widowed)	159	86.9	33	50.0	21	39.6	13	50.0	
Presence of a child									<0.001
No	165	90.2	39	59.1	22	41.5	3	11.5	
Yes	18	9.8	27	40.9	31	58.5	23	88.5	<0.001
Presence of adult family members who needs nursing care									<0.001
No	177	96.7	63	95.5	44	83.0	21	80.8	
Yes	6	3.3	3	4.6	9	17.0	5	19.2	
Hours of weekly domestic chores, median (25%, 75%)	1 (1, 3)		2 (1, 4)		4 (2, 5)		2 (1.5, 3)		<0.001
Hours of weekend domestic chores, median (25%, 75%)	2 (1, 4)		4 (2, 10)		5 (4, 11)		3.75 (2, 5)		<0.001
Work-family conflict									0.610
Low	50	27.3	18	27.3	11	20.8	9	34.6	
High	133	72.7	48	72.7	42	79.3	17	65.4	
Gender division of labor									<0.001
Disagree	61	33.3	30	45.5	26	49.1	19	73.1	
Agree	122	66.7	36	54.6	27	50.9	7	26.9	
Career experience year, median (25%, 75%)	3 (2, 5)		12 (10, 14)		20 (16, 22)		27.5 (18, 30)		<0.001
Average daily working hour, median (25%, 75%)	9 (8, 10)		8.75 (8, 10)		9 (8, 10)		8.9 (8,10)		0.792
Number of nights worked per month, median (25%, 75%)	4 (3, 5)		3 (0, 5)		2.5 (0, 4)		2.5 (2, 4)		<0.001
Job strain									0.113
Low strain	78	42.6	19	28.8	25	47.2	13	50.0	
High strain	105	57.4	47	71.2	28	52.8	13	50.0	
Job support									0.099
Low support	79	43.2	39	59.1	28	52.8	15	57.7	
High support	104	56.8	27	40.9	25	47.2	11	42.3	
Copenhagen burnout inventory, mean ± SD									
Work-related	48.2 ± 19.74		43.7 ± 16.20		40.1 ± 20.11		32.1 ± 19.05		0.001
Personal	54.7 ± 23.54		53.7 ± 16.44		48.6 ± 22.09		35.4 ± 20.87		0.029
Client-related	39.9 ± 22.4		36.3 ± 16.31		36.2 ± 20.44		35.4 ± 17.37		0.749
Intention to leave									0.014
Yes	146	79.8	49	74.2	33	62.3	15	57.7	
No	37	20.2	17	25.8	20	37.7	11	42.3	

According to age group category, “intention to leave” and all variables were statistically assessed by chi-square tests for categorical variables and one-way analysis of variance for contentious variables.

**Table 2:** Variables associated with “intention to leave”.

Variables	Intention to Leave				
	Yes		No		*p*
	*N* = 243	%	*N* = 85	%	
Age group					0.006
20s	146	60.1	37	43.5	
30s	49	20.2	17	20.0	
40s or older	48	19.8	31	36.5	
Marital status					0.906
Married	76	31.3	26	30.6	
Single (Including Divorced or Widowed)	167	68.7	59	69.4	
Presence of a child					0.005
No	180	74.1	49	57.7	
Yes	63	25.9	36	42.4	
Presence of adult family members who needs nursing care					0.333
No	224	92.2	81	95.3	
Yes	19	7.8	4	4.7	
Hours of weekly domestic chores, median (25%, 75%)	2 (1, 3)		2 (1, 3)		0.560
Hours of weekend domestic chores, median (25%, 75%)	3 (2, 5)		4 (2, 5)		0.581
Work-family conflict					0.140
Low	60	24.7	28	32.9	
High	183	75.3	57	67.1	
Gender division of labor					0.003
Disagree	89	36.6	47	55.3	
Agree	154	63.4	38	44.7	
Career experience as present worksite, median (25%, 75%)	6 (3, 13)		8 (3, 20)		0.105
Average daily working hour, median (25%, 75%)	9 (8, 10)		8 (8, 10)		0.038
Number of nights worked per month					0.097
Less than 4	109	44.9	47	55.3	
4 or more	134	55.1	38	44.7	
Job strain					0.010
Low strain	90	37.0	45	52.9	
High strain	153	63.0	40	47.1	
Job support					<0.0001
Low support	137	56.4	24	28.2	
High support	106	43.6	61	71.8	
Copenhagen burnout inventory, mean ± SD					
Work-related	49.1 ±19.10		32.9 ±15.38		<0.0001
Personal	56.6 ±21.85		40.8 ± 18.33		<0.0001
Client-related	41.9 ± 20.73		27.5 ± 16.19		<0.0001

According to “intention to leave”, all variables were evaluated by chi-square tests for categorical variables and one-way analysis of variance for continuous variables. Number of nights worked per month was divided into binary variable with a median.

**Table 3:** Factors associated with “intention to leave”—univariate logistic regression model.

Variables		Intention to Leave		
		Odds Ratio	95% CI	
			Lower	Upper
Age group				
20’s		2.55	1.43	4.54
30’s		1.86	0.91	3.80
40’s or older		1.00		
Marital status				
Married		1.00		
Single (including divorced or widowed)		0.97	0.57	1.65
Presence of a child				
No		1.00		
Yes		0.48	0.28	0.80
Presence of adult family members who needs nursing care				
No		1.00		
Yes		1.72	0.57	5.20
Weekly domestic labor				
More		0.75	0.46	1.25
Less		1.00		
Weekend domestic labor				
More		0.85	0.51	1.40
Less		1.00		
Work-family conflict				
Low		1.00		
High		1.50	0.88	2.57
Gender division of labor				
Disagree		1.00		
Agree		2.14	1.30	3.53
Career experience as present worksite				
More		0.86	0.52	1.44
Less		1.00		
Averaged daily working hours				
More		1.16	0.98	1.37
Less		1.00		
Number of nights worked per month				
Less than 3		1.00		
4 or more		1.52	0.93	2.50
Job strain				
Low strain		1.00		
High strain		1.91	1.16	3.15
Job support				
Low support		1.00		
High support		0.30	0.18	0.52
Copenhagen burnout inventory				
10	Work-related	1.67	1.42	1.97
10	Personal	1.42	1.25	1.62
10	Client-related	1.51	1.30	1.76

A logistic regression model was used to investigate influence of each factor on intention to leave. Odds ratio (OR) was computed along with 95% confidence intervals (95% CIs). Weekly domestic labor, weekend domestic labor, career experience as the present worksite, and averaged daily working hours were divided by its median.

**Table 4:** Stepwise multivariable regression model for factors associated with “intention to leave” adjusting for each domain of the Copenhagen Burnout Inventory.

Variable		Work-Related Burnout			Personal Burnout			Client-Related Burnout		
		Odds Ratio	95% CI		Odds Ratio	95% CI		Odds Ratio	95% CI	
			Lower	Upper		Lower	Upper		Lower	Upper
Age										
20s		2.37	1.22	4.61	2.85	1.49	5.46	3.31	1.72	6.38
30s		1.52	0.69	3.35	1.49	0.69	3.24	1.90	0.86	4.16
40s or older		1.00			1.00			1.00		
Job strain										
Low strain		1.00			1.00			1.00		
High strain		0.92	0.51	1.67	0.88	0.48	1.61	1.02	0.57	1.82
Job support										
Low support		1.00			1.00			1.00		
High support		0.33	0.18	0.60	0.28	0.16	0.52	0.30	0.16	0.55
Copenhagen burnout inventory										
10	Work-related	1.58	1.32	1.89						
10	Personal				1.39	1.20	1.60			
10	Client-related							1.47	1.24	1.73

The result of the multi-variable logistic regression model for independent variables (work factors, personal factors and perception toward work-family conflict) were entered into the equation, using a stepwise method.
